# Neutron Activated Samarium-153 Microparticles for Transarterial Radioembolization of Liver Tumour with Post-Procedure Imaging Capabilities

**DOI:** 10.1371/journal.pone.0138106

**Published:** 2015-09-18

**Authors:** Nurul Ab. Aziz Hashikin, Chai-Hong Yeong, Basri Johan Jeet Abdullah, Kwan-Hoong Ng, Lip-Yong Chung, Rehir Dahalan, Alan Christopher Perkins

**Affiliations:** 1 University of Malaya Research Imaging Centre, Faculty of Medicine, University of Malaya, Kuala Lumpur, Malaysia; 2 Department of Biomedical Imaging, Faculty of Medicine, University of Malaya, Kuala Lumpur, Malaysia; 3 Department of Pharmacy, Faculty of Medicine, University of Malaya, Kuala Lumpur, Malaysia; 4 Medical Technology Division, Malaysian Nuclear Agency, Kajang, Selangor, Malaysia; 5 Radiological and Imaging Sciences, Medical Physics and Clinical Engineering, Medical School, University of Nottingham, Nottingham, United Kingdom; Icahn School of Medicine at Mount Sinai, UNITED STATES

## Abstract

**Introduction:**

Samarium-153 (^153^Sm) styrene divinylbenzene microparticles were developed as a surrogate for Yttrium-90 (^90^Y) microspheres in liver radioembolization therapy. Unlike the pure beta emitter ^90^Y, ^153^Sm possess both therapeutic beta and diagnostic gamma radiations, making it possible for post-procedure imaging following therapy.

**Methods:**

The microparticles were prepared using commercially available cation exchange resin, Amberlite IR-120 H^+^ (620–830 μm), which were reduced to 20–40 μm via ball mill grinding and sieve separation. The microparticles were labelled with ^152^Sm via ion exchange process with ^152^SmCl_3_, prior to neutron activation to produce radioactive ^153^Sm through ^152^Sm(n,γ)^153^Sm reaction. Therapeutic activity of 3 GBq was referred based on the recommended activity used in ^90^Y-microspheres therapy. The samples were irradiated in 1.494 x 10^12^ n.cm^-2^.s^-1^ neutron flux for 6 h to achieve the nominal activity of 3.1 GBq.g^-1^. Physicochemical characterisation of the microparticles, gamma spectrometry, and *in vitro* radiolabelling studies were carried out to study the performance and stability of the microparticles.

**Results:**

Fourier Transform Infrared (FTIR) spectroscopy of the Amberlite IR-120 resins showed unaffected functional groups, following size reduction of the beads. However, as shown by the electron microscope, the microparticles were irregular in shape. The radioactivity achieved after 6 h neutron activation was 3.104 ± 0.029 GBq. The specific activity per microparticle was 53.855 ± 0.503 Bq. Gamma spectrometry and elemental analysis showed no radioactive impurities in the samples. Radiolabelling efficiencies of ^153^Sm-Amberlite in distilled water and blood plasma over 48 h were excellent and higher than 95%.

**Conclusion:**

The laboratory work revealed that the ^153^Sm-Amberlite microparticles demonstrated superior characteristics for potential use in hepatic radioembolization.

## Introduction

Transarterial radioembolization (TARE) is a minimally invasive procedure involving intravascular administration of radioembolic microparticles for the treatment of liver lesions. The most common type of liver malignancy is hepatocellular carcinoma (HCC) which is the second most common cause of death from cancer worldwide [1]. HCC is often diagnosed at the later stages when other curative approaches are no longer feasible [2]. There are two commercially available radioembolic agents for TARE; glass-based TheraSphere^®^ (Nordion, Canada) and resin-based SIR-Spheres^®^ (SIRTex, Australia) microspheres, which both were approved by the United States Food and Drug Administration (FDA) as medical devices. TheraSphere^®^ was approved under Humanitarian Device Exemption (HDE) for radiation treatment, or as neoadjuvant to surgery or transplantation, for HCC patients [3]. On the other hand, SIR-Spheres^®^ was approved under Premarket Approval (PMA) for liver metastases secondary to colorectal cancer [4]. They both act as permanent brachytherapy implants localised at the target tumour site until fully decay, delivering all the radiation *in situ*.

Both agents comprise of Yttrium-90 (^90^Y), which is commonly produced in a Strontium-90 (^90^Sr) generator.^90^Sr is obtained from nuclear fission of Uranium-235 (^235^U) fuel in a nuclear reactor. ^90^Y is rarely produced via neutron activation due to very low thermal neutron activation cross-section of ^89^Y. The various stages involved in the production of ^90^Y, have resulted in the high cost and limited supply of the radionuclide. Since ^90^Y is a pure beta emitter, the distribution of ^90^Y-microspheres after TARE procedure is difficult to be verified. Gamma imaging using macro-aggregated albumin (MAA) labelled with Technetium-99m (^99m^Tc) has been used prior to the treatment for lung shunting quantification, which provides reasonable information on the microspheres distribution within the liver and lungs. However, this method does not accurately reflect the intrahepatic distribution of the microspheres, due to resolution and partitioning dissimilarities between ^99m^Tc and ^90^Y images, as a result of different physical characteristics and number of particles infused [5,6]. Although the infused microspheres can be followed via Bremsstrahlung imaging, this technique is rather challenging and requires special procedures and post-processing skills. Also, the images were very low in spatial resolution due to a wide spectrum of scatter energies produced, as a result of beta attenuation. The importance of obtaining high resolution images that resembles the true biodistribution of the microparticles is that, it helps in accurately determining the region of extrahepatic microparticles deposition. This is to prepare the clinicians for any complications, i.e. hepatic dysfunction, biliary sequelae, radiation pneumonitis, gastroenteritis and acute pancreatitis [7], and also to evaluate the treatment efficacy.

Radionuclides which emit both therapeutic beta and diagnostic gamma energies would be ideal for “theranostic” (therapy plus diagnostic) application. An ideal theranostic radionuclide should has optimum physical half-life, suitable linear energy transfer (LET) and range in tissues, high ratio of non-penetrating to penetrating radiation, short lived or stable daughter, good and selective concentration with prolonged retention in tumour, and minimum uptake by normal tissue [8]. Neutron activation is preferred in radionuclide production due to wide availability of nuclear reactors and relatively simpler process. It also permits lower radiation exposures to the personnel since, the preparation of the nuclide labelled embolic agent is being carried out prior to neutron activation.

Samarium-153 (^153^Sm) is potentially suitable as an alternative to ^90^Y in liver cancer treatment. It has been widely used for palliative pain treatment of bone metastatic patients as well as radiation synovectomy of the knees, however its therapeutic potential has not been fully utilised for other cases. The imaging properties of ^153^Sm has been proven feasible for gastrointestinal scintigraphy [9]. The main advantages of ^153^Sm are its optimal half-life (1.93 days), relatively high thermal neutron activation cross-section (210 barns) and diagnostic gamma energy of 103 keV [10,11], which can be easily distinguished via energy windowing hence, images with high spatial resolution and minimal noises can be obtained following each procedure. Although its beta energy is about 2.8 times lower than ^90^Y, this can always be compensated by administering higher activity of ^153^Sm to deliver complementary therapeutic dose to the tumour, which can be easily achieved due to its high cross-section value.

One of the important features of radioembolic agents for TARE is the particle size, which should range between 20–40 μm. Larger microparticles would not reach the capillary bed while the smaller microparticles may pass through the tumour capillaries and reach the neighbouring organs especially the lungs [12]. Microparticles with resistivity to physical heat and body chemicals, near plasma density, biocompatible, non-biodegradable and easily labelled with radionuclides are highly preferred. Due to these reasons, ion exchange resins offer the best properties as radioembolic agents. They are useful as carriers for medicinal materials and in slow release applications [13], and are known to be able to withstand extreme pH in gastric fluid while retaining its function at its best.

This research aimed to develop a safer and cheaper alternative microparticles via simpler methods for TARE, with ability of post-procedure imaging. The microparticles were prepared via ion exchange with ^152^SmCl_3_ salt, followed by neutron activation to generate radioactive ^153^Sm. The physicochemical characterisation, determination of radionuclide impurities, and radiolabelling efficiency studies were carried out and explained in detail in this article.

## Materials and Methods

### Preparation of ^152^Sm-Amberlite microparticles

**Fig 1:**
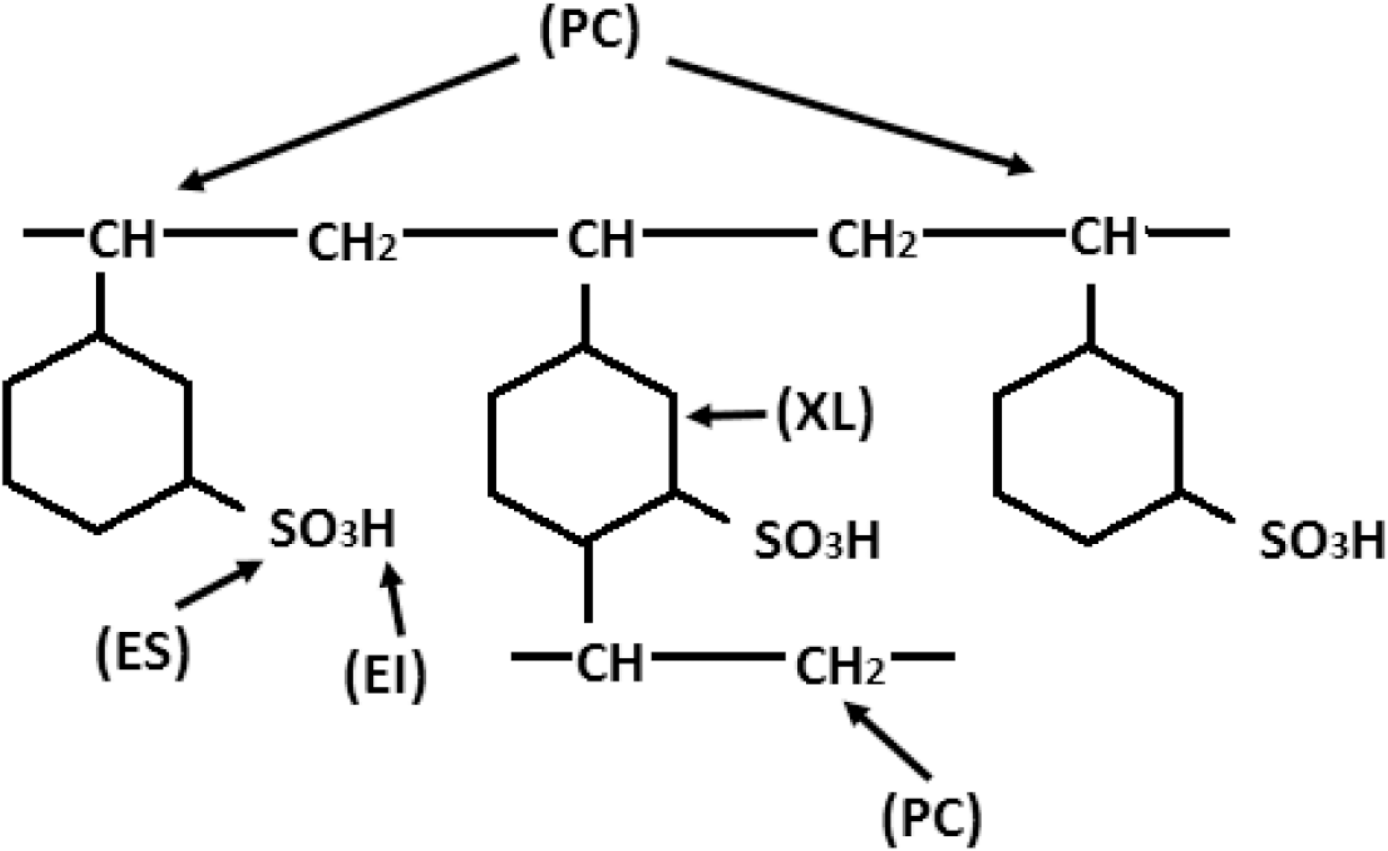
Chemical structure of Amberlite IR-120 H^+^ (PC: polymer chain, XL: cross-link, ES: exchange site, EI: exchangeable ion).

A commercially available styrene-divinylbenzene (DVB) ion exchange resin with sulphonic acid group, Amberlite IR-120 H^+^ was obtained from Fluka GmbH (Buchs, Switzerland). Its typical properties and chemical structure are shown in Table 1 and Fig 1, respectively. Samarium (III) chloride hexahydrate (^152^SmCl_3_·6H_2_O; molecular weight 364.81 g.mol^-1^; isotopic abundance 26.7%) with assay purity ≥ 99% was obtained from Aldrich Chemical Co. (Wisconsin, USA).

**Table 1:** Typical properties of Amberlite IR-120 H^+^ as per manufacturer [14,15].

**Matrix**	Styrene-divinylbenzene copolymer
**Functional group**	Sulphonic acid
**Ionic form**	H^+^
**Cross-linkage (%)**	8
**Moisture (%)**	53–58
**Cation-exchange capacity (meq/g)**	5.35 (dry)
**Total exchange capacity (eq/l)**	1.8 (by wetted bed volume)
**Particle size (μm)**	620–830
**Operating pH**	0–14

The Amberlite IR-120 resin was dried in a laboratory oven at 70°C for 12 h. In order to meet the desired size for intraarterial administration to the hepatic artery, the dried resin was ground using a grinding planetary ball mill machine (XQM-(2–6)L, ChangSha LangFeng Metallic Material Ltd., China) at 200 rpm for approximately 5 h. The resin powder was subsequently sieved using a mechanical sieve shaker (AS 200 Analytical Sieve Shaker, Retsch GmbH, Haan, Germany) attached with 20 and 40 μm wire mesh stainless steel test sieves (Endecotts Ltd., London, UK).

1 g of SmCl_3_.6H_2_O was dissolved in 10 ml distilled water. 5 g of the Amberlite resin powder was poured into the SmCl_3_ solution and stirred thoroughly for 5 min to allow binding of the Sm^3+^ ions to the resin. The ^152^Sm-Amberlite resin was then washed with distilled water, using Büchner funnel filtration system, to remove the Cl^-^ ions and any unbound Sm^3+^ off the resin. Finally, the formulation was dried in the oven at 70°C for 12 h. The chemical equations for the preparation processes are listed as follows:

Dissolution of Samarium salt:

$$SmCl_{3}.6H_{2}O_{\left(s\right)}+H_{2}O_{\left(l\right)}\to Sm^{3+}{}_{\left(aq\right)}+3Cl^{-}{}_{\left(aq\right)}+7H_{2}O_{\left(l\right)}$$ (1)

Labelling of Samarium onto resin via ion exchange process:

$$3R-SO_{3}H_{\left(s\right)}+\;Sm^{3+}{}_{\left(aq\right)}+\;3Cl^{-}{}_{\left(aq\right)}\to {\left(R-SO_{3}\right)}_{3}Sm_{\left(s\right)}+\;3HCl_{\left(aq\right)}$$ (2)

Refinement of Sm-Amberlite microparticles:

$${\left(R-SO_{3}\right)}_{3}Sm_{\left(s\right)}+\;3HCl_{\left(aq\right)}\overset{+H_{2}O_{\left(l\right)}}{\underset{filtration}{\to }}{\left(R-SO_{3}\right)}_{3}Sm_{\left(s\right)}+\;H_{2}O\overset{oven}{\underset{dry}{\to }}{\left(R-SO_{3}\right)}_{3}Sm_{\left(s\right)}$$ (3)

where: *R* = [*CH*_2_ *CH*(*C*_6_ *H*_5_)]×[*CH*_2_ *CH*[*C*_6_ *H*_4_(*CHCH*_2_)]]_*y*_

### Neutron activation

The ^152^Sm-Amberlite microparticles were sent for neutron activation at Malaysian Nuclear Agency (MNA). The TRIGA PUSPATI Reactor (RTP) (Triga Mark II, General Atomics, California, USA) is a pool type, 7 m high aluminium tank surrounded by high density concrete, regularly operates at 750 kW power level. The solid fuel element used is of enriched uranium (20% weight, ^235^U), homogenously combined with zirconium-hydride moderator.

The microparticles were sealed in individual polyethylene vial and placed into a polyethylene ampoule for neutron activation. Two methods of neutron activation via pneumatic transfer system (PTS) and rotary specimen rack (RR), were studied to achieve high therapeutic activity (3 GBq) of ^153^Sm. The protocols for both neutron activation methods are shown in Table 2. The irradiation time can be estimated using Eq 4.

$$A_{t}=\sigma_{act}\varphi \;N\;(1\;–\;e^{-}{}^{\lambda t})$$ (4)

where;

A_t_ = activity (Bq)

σ_act_ = thermal neutron activation cross-section (barns = 10^−24^ cm^2^)

φ = neutron flux (n.cm^-2^.s^-1^)

N = number of parent atoms = (m / w) x θ x 6.023 x 10^23^

m = mass of element in the sample

w = atomic weight of element

θ = isotopic abundance

λ = decay constant (s^-1^)

t = irradiation time (s)

**Table 2:** Neutron activation protocols to achieve ^153^Sm activity of 3 GBq.

Method	PTS	RR
**Thermal neutron flux, θ**_**th**_ **(n.cm** ^**-2**^ **.s** ^**-1**^ **)**	4.813 x 10^12^	1.494 x 10^12^
**Irradiation time**	5 min	6 h
**Location in the reactor**	Near to the core	Peripheral to the core
**Sample entrance and exit**	Automatic	Manual

After activation, the samples were left for 48 h to allow for complete decay of the short-lived contaminant radionuclides (i.e. ^38^Cl). The activities of the samples were then measured using a dose calibrator (CRC 25R, Capintec, New Jersey, USA). The activity per microparticle was calculated by dividing the sample activity with the estimated number of microparticles in the samples.

### Gamma spectrometry

After 48 h, gamma spectrometry was carried out for each sample to determine the presence of radionuclide impurities, especially the long-lived radionuclides. A coaxial, p-type hyper-pure germanium detector (Canberra, Meriden, USA) and gamma spectrum analysis software (Genie^TM^ 2000 Ver. 3.2, Canberra, Meriden, USA) were used. Each sample was counted for 5 min live time at a calibrated distance. Radioactive elements that corresponded to the significant energy peaks were identified.

### Physicochemical characterisation of ^153^Sm-Amberlite microparticles

Fourier Transform Infrared (FTIR) spectroscopy (600–4000 cm^-1^ range) on the Amberlite resins were carried out using a FTIR spectrometer (Nicolet 6700, Thermo Fisher Scientific Inc., Massachusetts, USA) to investigate chemical structure changes due to mechanical grinding process. FTIR spectra of fresh Amberlite resin beads, resin microparticles after grinding and sieving processes, resin microparticles after labelling with SmCl_3_ salt, and resin microparticles after 6 h continuous neutron activation were compared and the differences between major peaks were investigated. In addition, the FTIR spectrum of SmCl_3_ salt was also obtained for comparison.

Field Emission Scanning Electron Microscopy (FESEM) and Energy Dispersive X-ray (EDX) spectroscopy were carried out on the Sm-Amberlite resins before and after neutron activation using a FESEM system (Quanta FEG 250, FEI, Oregon, USA), for structural observations and validation of chemical compositions, respectively. The images obtained were analysed using particle analysis in ImageJ software (version 1.45s, US National Institutes of Health, Maryland, USA). The mean diameter and distribution of the particle sizes were determined for both samples.

The particle density, ρ_s_ and total pore volume (TOPV) of ^153^Sm-Amberlite microparticles were measured using helium gas pycnometer (AccuPvc II 1340, Micromeritics Ins. Corp., Georgia, USA) at standard room temperature of 25°C. From the measured values, the porosity (%) of the microparticles was calculated using Eq 5.

$$Porosity\;\left(\%\right)\;=\;\left[TOPV/\;\left(\left(1\;/\;\rho_{s}\right)+\;TOPV\right)\right]\;\times \;100\;\%$$ (5)

The ρ_s_ value was then incorporated into Eq 6 [16], to estimate the particle concentration (particles.ml^-1^) of the microparticles suspended in 0.9% saline solution.

$$Particle\;concentration\;\left(particles.ml^{-1}\right)=6\;\rho_{f}/\left[\pi D_{p}{}^{3}\left(\rho_{f}+\left(\rho_{s}/C\right)-\;\rho_{s}\right)\right]$$ (6)

where;

C = mass fraction (% w/w)

D_*p*_ = mean diameter of the particles (cm)

ρ_*f*_ = density of the solvent (g.cm^-3^)

ρ_*s*_ = particle density (g.cm^-3^).

The viscosity, η_o_ of 2.5% w/v microparticles in saline suspension was measured at 37°C, using DV-II Pro EXTRA viscometer (Brookfield Engineering Labs Inc., Massachusetts, USA). The value was then incorporated into Stokes’ Law (Eq 7) to study the sedimentation rate (settling velocity) of the suspension.

$$V_{sed}=\left[g\;D_{p}{}^{2}\left(\rho_{s}–\;\rho_{f}\right)\right]/\;18\eta_{o}$$ (7)

where;

V_sed_ = sedimentation rate (cm.s^-1^)

g = gravitational acceleration constant (981 cm.s^-2^)

η_o_ = dynamic viscosity of the fluid (P = g.cm^-1^.s^-1^)

### Radiolabelling efficiency and determination of optimum formulation

1 g of SmCl_3_.6H_2_O was labelled to 1, 2, 3, 4, 5, and 6 g of the resin to determine optimum formulation with best labelling efficiency. All samples were activated via PTS for 5 min. Each sample was equally separated into three 10 ml test tubes followed by addition of 10 ml distilled water. The samples were mixed using a roller mixer (Movil-Rod, J.P. Selecta, Barcelona, Spain) at 50 rpm for 1 h. Next, the samples were centrifuged at 1200 rpm for 5 min. 1 ml of supernatant was pipetted from each tube and transferred into gamma assay tubes. These steps were repeated until a total of 8 ml supernatants were obtained from each sample within 48 h. All supernatant samples were assayed using gamma scintillation counter (2470 Wizard2, PerkinElmer Inc., Massachusetts, USA). All steps were repeated in human blood plasma which was obtained from Department of Transfusion Medicine, University Malaya Medical Centre (UMMC), Malaysia [17]. Medical ethics approval was not required with reference to Scope 2.1.3, Standard Operating Procedure (SOP) of UMMC Medical Ethics Committee, since no personal identity information of the donor was acquired for this research [18,19]. Hence, donor consent was also not required due to anonymity of the sample. Labelling efficiency of each formulation was calculated using Eq 8 [20].

$$Retainedactivity\;\left(\%\right)=\left[\left(A_{sus}-\;A_{\text{sup}}\right)/\;A_{sus}\right]\;\times \;100\;\%$$ (8)

where;

A_sus_ = Activity of resin suspension before each extraction of 1 ml supernatant

A_sup_ = Activity of 1 ml supernatant

## Results

### Determination of neutron activation protocol

Despite the convenience of sample delivery into the nuclear reactor and three times higher neutron flux than the RR method, the automated delivery system of the PTS method only allowed a maximum of 5 min irradiation per sample at one time. The specific activity per g of the ^153^Sm-Amberlite microparticles after 5 min activation via PTS was 0.148 ± 0.004 GBq, far lower than the aimed therapeutic activity. Following 6 h activation via RR was able to achieve 3.104 ± 0.029 GBq of ^153^Sm activity. Hence, the RR method was shown to be feasible for the production of therapeutic ^153^Sm. The specific activity per microparticles following 6 h neutron activation was 53.855 ± 0.503 Bq.

### Gamma spectrometry for determination of radionuclide impurities

The most dominant photopeak observed was at 103.1 ± 0.2 keV, which is the principal gamma energy emitted by ^153^Sm. The other gamma energy emitted by ^153^Sm was 69.4 ± 0.2 keV, which was also revealed in the gamma spectrometry. Two other significant peaks appeared consistently in all samples were 40.7 ± 0.2 and 46.5 ± 0.2 keV. These peaks resulted from the K-shell characteristic X-rays following radioactive decay. No radionuclide impurity was observed in the ^153^Sm-Amberlite samples.

### Physicochemical characterisation of ^153^Sm-Amberlite microparticles

**Fig 2:**
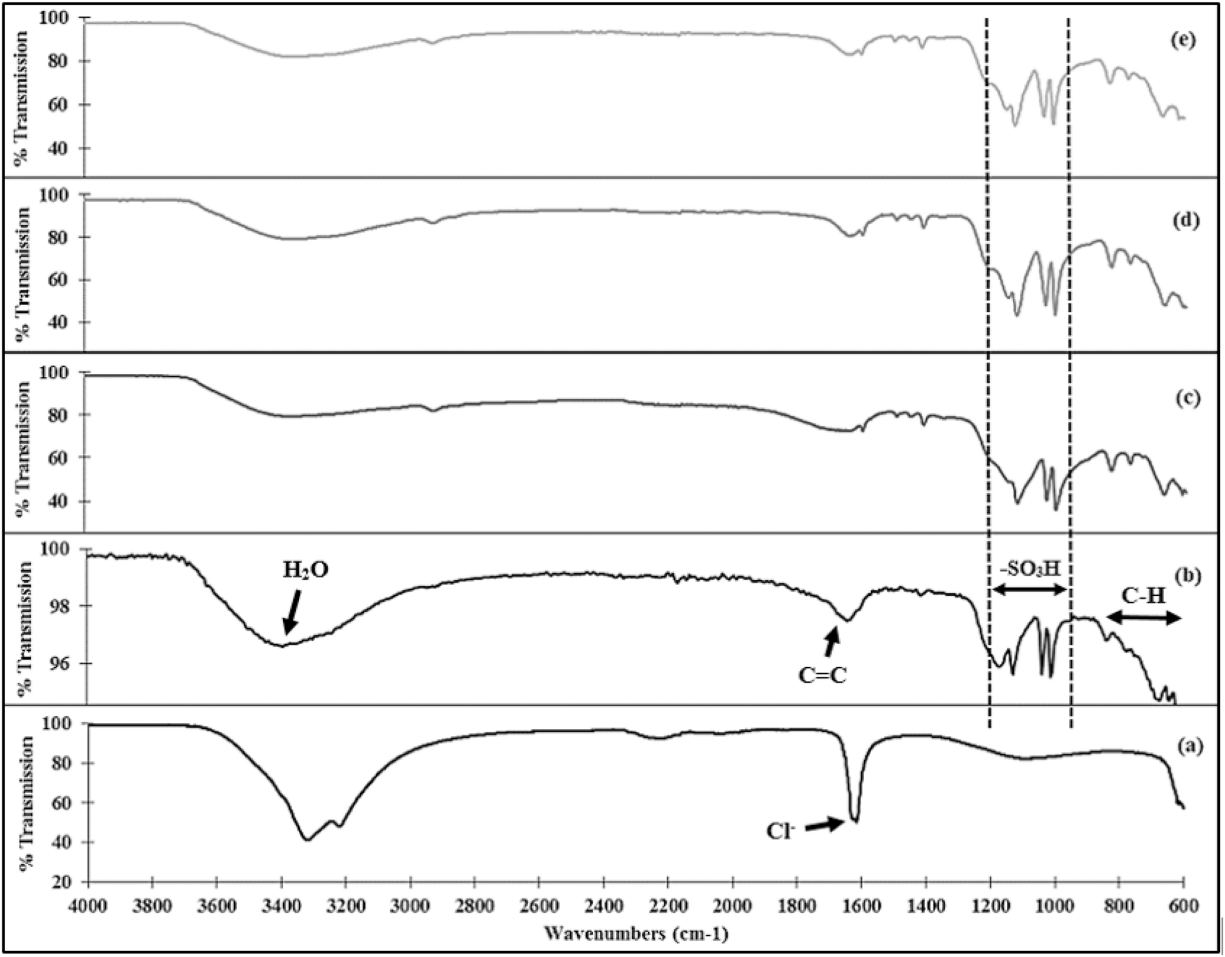
FTIR spectra of (a) SmCl_3_.6H_2_O salt, (b) Fresh Amberlite IR-120 H^+^ beads, (c) Amberlite IR-120 H^+^ ground and sieved to size 20–40 μm, (d) Amberlite microparticles labelled with SmCl_3_.6H_2_O salt, and (e) ^153^Sm-Amberlite microparticles after 6 h neutron activation.

The comparison between spectra in different production stages alongside SmCl_3_ spectrum is shown in Fig 2(a)–2(e). FTIR spectrum of the fresh Amberlite IR-120 H^+^ resin in Fig 2(b) was compared to a FTIR spectrum of the same resin from a previous study [21]. As compared to the other spectra, the water content within the samples decreased following size reduction, labelling and neutron activation. As shown in Fig 2(b)–2(e), the functional sulphonic group within the ion exchange resins were still present at each production stages. There were no major differences observed between those peaks.

**Fig 3:**
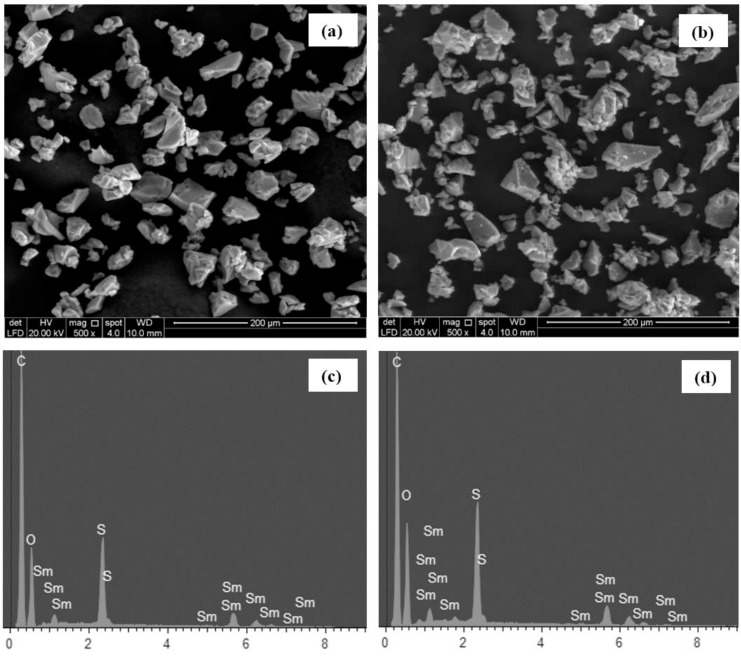
FESEM images of Sm-Amberlite microparticles (a) before and (b) after 6 h neutron activation with their corresponding EDX spectra (c) and (d), respectively.

The FESEM images of the microparticles before and after neutron activation are shown in Fig 3(a) and 3(b). The microparticles were observed to be irregular in shape. Following neutron activation, increase amount of smaller fragments were observed, however no major changes in the overall shapes of the microparticles. The EDX spectra of the microparticles are shown in Fig 3(c) and 3(d). Both spectra confirmed the composition of the resins which comprised of C, O, Sm, S and H, which the later cannot be detected in EDX analysis due to atomic number < 6. The elemental weight fractions of each samples indicated about 7% of Sm was present, hence each 1 g of the ^153^Sm-Amberlite microparticles consisted of 70 mg ^153^Sm.

**Fig 4:**
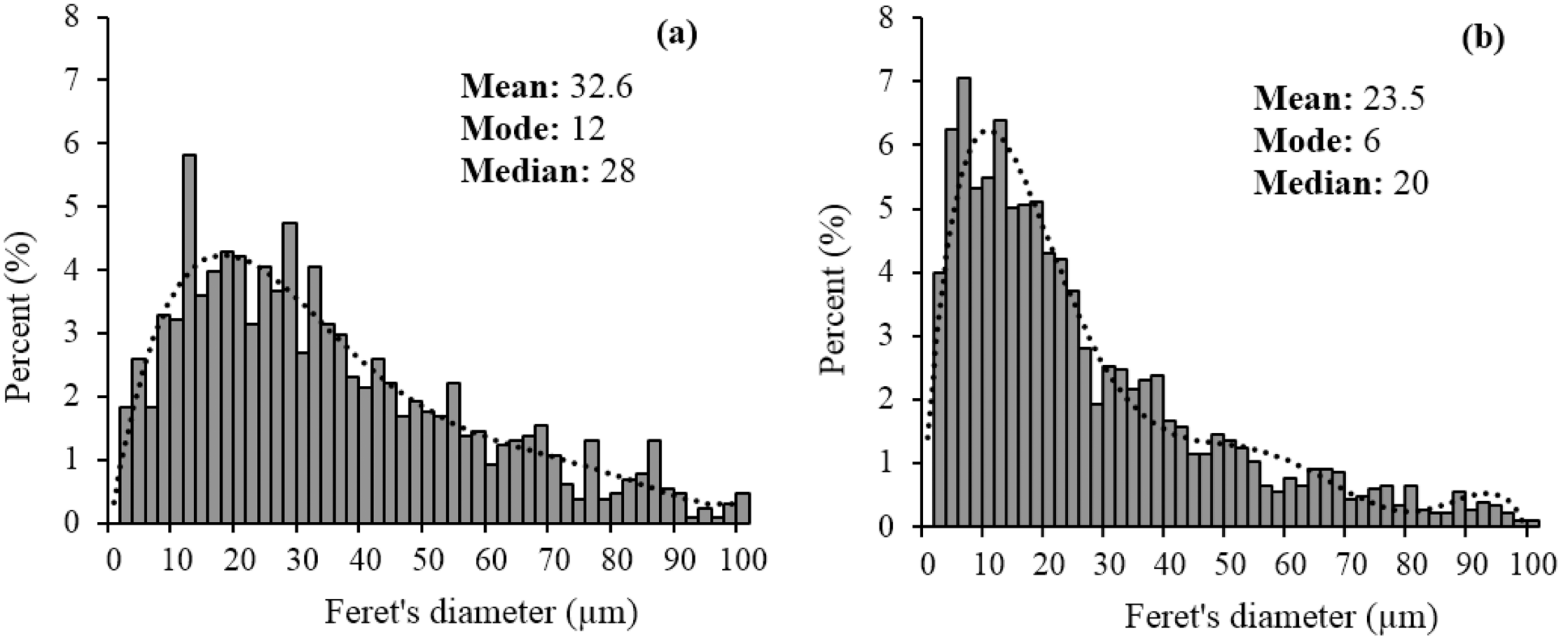
Particle size distribution of Sm-Amberlite microparticles (a) before and (b) after 6 h neutron activation.

The particle size distributions of both samples are shown in Fig 4(a) and 4(b). Since, the microparticles were non-spherical, the Feret’s diameter measurements were used. The mean diameter of the particles before and after 6 h neutron activation were 32.6 ± 2.1 μm and 23.5 ± 2.3 μm, respectively. Before neutron activation, 34.7 ± 2.6% of the microparticles were in the range of 20–40 μm, while 72.1 ± 4.9% were in the range of 10–60 μm. Following 6 h neutron activation, 28.0 ± 1.5% remained in the 20–40 μm range, while 62.9 ± 6.7% of the microparticles were in the range of 10–60 μm. The differences in size distributions of the microparticles were mainly contributed by the increase amount of fragments (< 10 μm) following neutron activation.

**Fig 5:**
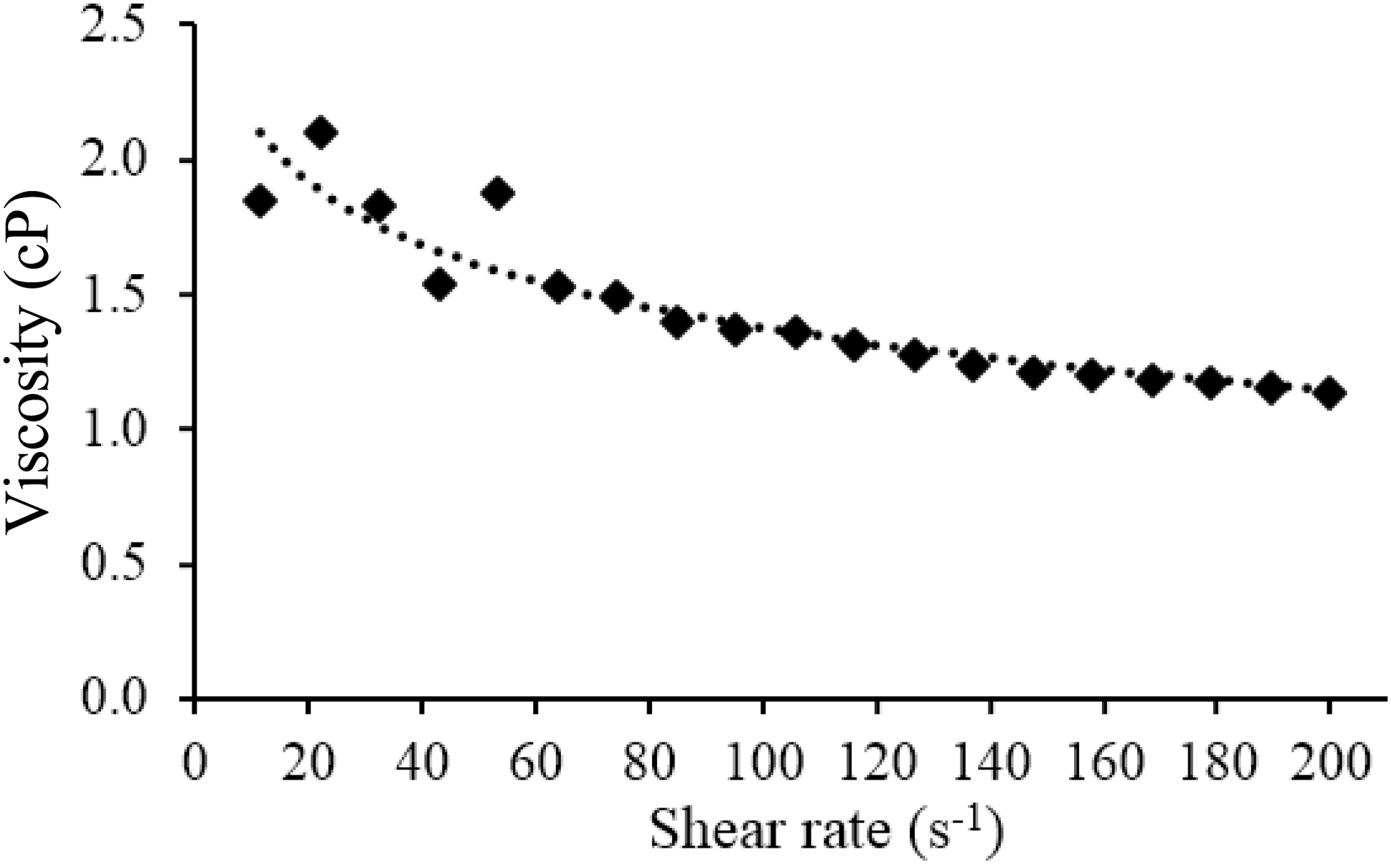
The viscosity of 2.5% w/v suspension of ^153^Sm-Amberlite in saline at 37°C with various shear rate.

The dynamic viscosities of the suspension at 37°C are shown in Fig 5. The differences in viscosity at various shear rates were small, hence the mean viscosity (1.43 ± 0.29 cP) was used to estimate the settling velocity of the suspension. The estimated settling velocity for the suspension was 0.033 ± 0.006 cm.s^-1^. The particle density and porosity of the microparticles were 2.538 ± 0.012 g.cm^-3^ and 61%, respectively. The comparisons between microparticles developed in this study as compared to previous published studies are tabulated in Table 3.

**Table 3:** Physical characteristics of previously produced radionuclide labelled microparticles for TARE [10,22–25] as compared to this study.

Manufacturer	TheraSphere	SIR-Spheres	This study	Mumper et al. 1991	Hruby et al. 2011	Poorbaygi et al. 2011	Hafeli et al. 1999
**Radionuclide**	^90^Y	^90^Y	^153^Sm	^166^Ho	^177^Lu	^90^Y (^177^Lu)	^186^Re/ ^188^Re
**T**_**1/2**_ **(h)**	64.08	64.08	46.32	26.80	159.6	64.08 (159.6)	89.28/ 17.00
**Max Eᵦ- (keV) [% yield]**	2279.8 [99.98]	2279.8 [99.98]	807.6 [19.5]	1854.5 [48.2]	498.3 [79.3]	2279.8 [99.98]	2120.4 [71.7]
**Principal Eγ (keV) [% yield]**	None	None	103.2 [29.2]	80.6 [6.6]	208.4 [10.4]	208.4 [10.4]	155.0 [15.2]
**Matrix material**	Glass	Resin	Resin	PLLA	Polymer	Glass	Glass
**Size (μm)**	20–30	20–60	20–40	10–45	20–40	20–40	25–32
**Density (g cm** ^**-3**^ **)**	3.2	1.6	2.5	1.4	NP	3.3	2.9
**Activities (GBq) available per vial/ gram**	3, 5, 7, 10, 15, 20	3	3.1	25.9	NP	NP	NP
**Number of particles (million) per vial/ gram**	1.2, 2, 2.8, 4, 6, 8 respectively	40–80	57.6	132	NP	NP	NP
**Activity (Bq) per particles**	2,500	50	54	196	NP	NP	NP

NP: not provided; Eᵦ-: energy of beta emission; Eγ: energy of gamma emission

### Radiolabelling efficiency and determination of optimum formulation

**Fig 6:**
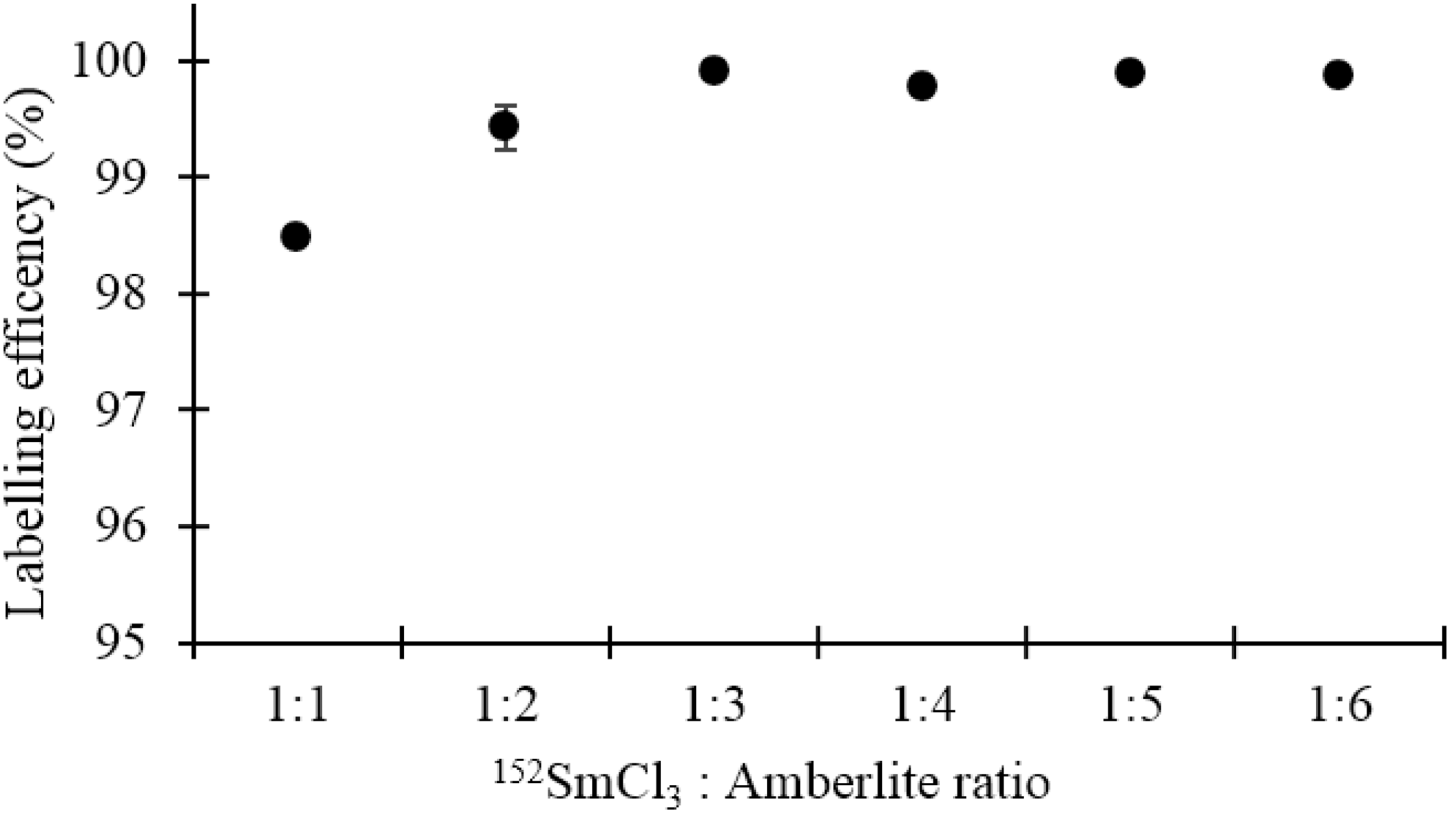
Labelling efficiency (%) of various formulations of ^153^Sm-Amberlite microparticles.

**Fig 7:**
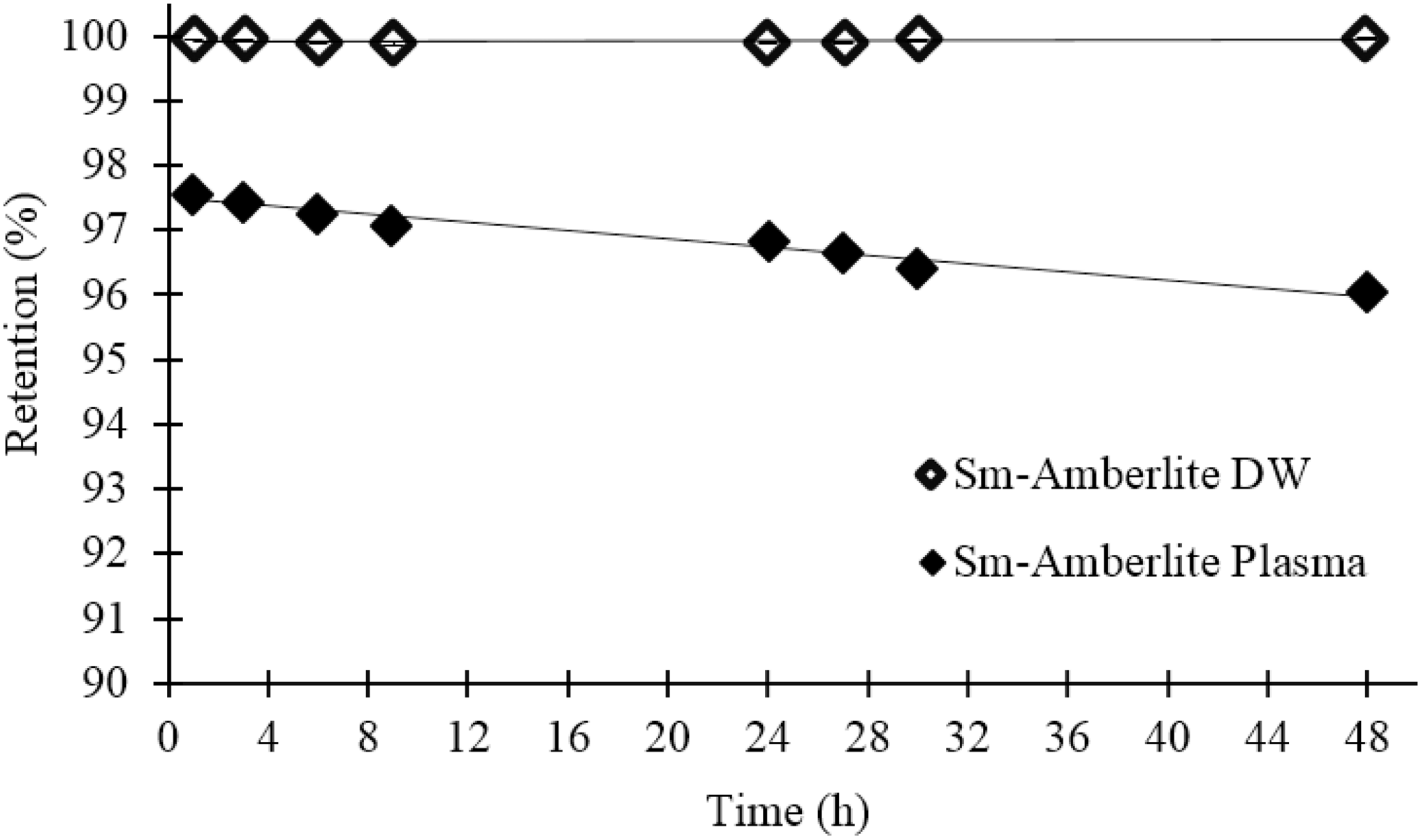
Retention (%) of ^153^Sm in Amberlite microparticles suspended in distilled water (DW) and blood plasma over 48 h.

Fig 6 shows the radiolabelling efficiency of the ^153^Sm-Amberlite microparticles. The labelling efficiency increased with resin: ^152^SmCl_3_ ratio until a plateau was reached. Therefore, the optimum ^152^SmCl_3_: resin ratio was found to be 1:3 with 99.92 ± 0.02% efficiency. The retention of ^153^Sm in the microparticles for an extended period of 48 h in both distilled water and blood plasma are shown in Fig 7. The retention in blood plasma was slightly lower than in distilled water, however it is still not less than 95% until 48 h.

## Discussion

Ion exchange resins were used in this study because of their commercial availability, safe and relatively easier labelling process. Due to their insoluble characteristic, resins will not be absorbed by the body thus, are very safe for use in medicinal products with limited side effects [26]. Amberlite IR-120 H^+^ was chosen due to its excellent labelling efficiency as reported in an earlier study [20], and also for having high selectivity towards Sm^3+^. The latter is due to the fact that, generally, the selectivity of ion exchange increases with increase of ion charge, and for similar charge ions, the selectivity increases for ions with higher atomic number.

This study successfully determined the neutron activation protocol for the ^153^Sm-Amberlite microparticles to achieve 3 GBq.g^-1^. The administered activities can be adjusted by manipulating the neutron activation time or the amount of ^153^Sm-Amberlite microparticles prescribed, according to individual patient dosing requirement. However, in terms of therapeutic efficacy, a large number of microparticles with medium specific activity is better as compared to lower number of microparticles with high specific activity. This is because, large number of microparticles will distribute the radiation dose more evenly, than those with lower number of microparticles with higher specific activity, even though the prescribed activity is the same. Hence, we recommend the manipulation of number of microparticles use rather than increasing the irradiation time.

There were no impurities present following 48 h post neutron activation, unlike other study where many impurities were produced, some with very long physical half-life [24]. This can be explained because, each of the elements (^12^C, ^1^H, ^32^S and ^16^O) present in the resin requires more than one neutron to be activated to radioactive isotopes. Thus, the probability of them being activated is relatively lower than that of ^152^Sm. Hence, the resin is chemically very stable and suitable for neutron activation.

The FTIR spectroscopy showed that harsh physical force applied on the resin beads during grinding process did not destroy the chemical structure of the resin especially its functional group. The FTIR spectra before and after neutron activation also indicated that high temperature during oven drying (70°C) and neutron activation (approximately 260°C) did not affect the chemical structure of the resins and its functional properties.

Following neutron activation, an increase in the number of smaller fragments were observed. The tendency to break could be influenced by the physical structure of the resin which is very jagged following size reduction. In order to overcome this problem, a spherical microparticles will be ideal since a spherical structure can withstand larger mechanical force due to a more compact structure. For the pre-irradiated microparticles, although a separation to obtain microparticles of 20–40 μm had been done via sieve analysis, presence of microparticles outside of this range were still observed. This can be explained by two reasons, one is since the microparticles did not possess a uniform structure, those with large Feret’s diameter might still passed the 40 μm sieve from its shorter edge. The second reason could be due to flocculation of smaller microparticles or overlapping of microparticles captured by the microscope. Thus, during image analysis, these microparticles were measured as one microparticle with very large diameter.

For the smaller range microparticles, the reason could be due to inadequate sieving as a result of difficulty of separating very fine microparticles and due to jagged physical structure, hence it requires longer time to be separated as compared to spherical particles. This can be improved by increasing the sieving time to ensure that the smaller microparticles can be removed or by using ultrasonic micro sieves to reduce time. The other reason could be due to noise produced during image analysis. Regardless, the microparticles in the range of 10–60 μm were still higher than 60% following neutron activation. Even though there were only 28% microparticles in the initial desired range of 20–40 μm, further improvements can be made with more spherical particles for improved particle strength and sieving efficiency, hence a more uniform microparticles distribution can be achieved within the desired range.

The viscosity of the suspension should be indistinguishable from normal saline, and relatively lower than blood viscosity (3 cP). The suspension was prepared in dilute form as it is during intraarterial administration to prevent blockage of the microcatheter. The settling velocity is relatively low due to reduced mean diameter following neutron activation, and should be better than that of glass microparticles, since the settling velocity is largely dependent on the diameter and particle density. Hence, the suspension is relatively stable. A lower settling rate and higher stability can be further achieved by using slightly higher viscosity solvent such as 5% dextrose solution, which is practiced by our institution for the administration of SIR-spheres.

The labelling efficiencies and retention of ^153^Sm were tested in both distilled water and blood plasma because they will be suspended in distilled water during packaging, and injected intra-arterially to the tumour during the treatment. ^153^Sm-Amberlite showed excellent efficiency of > 99% in distilled water to the extent of 48 h. This shows that the microparticles are very stable to be suspended in the medium during packaging, prior to intraarterial administration. Nonetheless, the efficiency dropped to approximately 97% immediately following suspension in blood plasma, followed by a gradual release to 96% efficiency at 48 h.

As compared to the other studies, the main advantages of the microparticles developed in this study are the ability to produce zero radionuclide impurities and also very high labelling efficiency following prolong neutron activation. Despite some breakage of the physical structure, the microparticles were still able to retain ^153^Sm for long period of 48 h and labelling efficiency higher than 95%. Unlike glass microparticles with high density, resin is a much better choice since it possess lower settling rate hence, will aid in easy microparticles delivery. The microparticles were able to provide great functionality despite long irradiation period which is not possible for Poly (L-lactic acid) (PLLA) or other biodegradable polymer [27].

In terms of the radionuclides used in previous studies, the production of Rhenium (Re) via neutron activation of the ReO_2_ [25] is not recommended, as this will produced both ^186^Re and ^188^Re, with the former decaying to an unstable daughter nuclide with long physical half-life. Also, for practicality, having half-lives of about 1 day or less might be a disadvantage for ^188^Re and Holmium-166 (^166^Ho). On the other hand, Luthetium-177 (^177^Lu) with half-life of 6.65 days will requires patients to be hospitalised, hence contributing to added cost. In terms of the therapeutic beta energy, ^166^Ho and ^188^Re provide high maximum beta energies, close to that of ^90^Y. However, since a major aim is to overcome the issue with post-procedure imaging, the energy of the gamma radiation is important in this respect. In this case, the gamma energy of ^166^Ho is considered to be relatively low thus, high resolution images might still not being achieved. ^177^Lu, on the other hand, possess the highest gamma energy amongst these radionuclides, although its therapeutic beta energy is very low. Another important aspects for radionuclides produced via neutron activation is the thermal neutron activation cross-section, σ_act_ (barns) of the target nuclide. ^176^Lu possess the highest value of 2050 barns [11], while both ^166^Ho and ^188^Re possess low cross-section values of less than 100 barns.

From the cost perspective, the methods of production and shipping have to be considered. ^90^Y is produced from a ^90^Sr-generator, a process that can be carried out “in house” with appropriate quality control measures to ensure the final radiochemical purity. The practicalities of this production method can be problematic in developing countries, where expertise is rather limited. The commercially produced ^90^Y-microspheres are more commonly used, but these need to be shipped internationally resulting in significantly higher costs due to the requirements for lead shielding and importation controls which vary greatly depending on the location of the end user. As an alternative, our technique requires ^153^Sm production through neutron activation of relatively low cost ^152^Sm salt, resulting in a product of high purity as demonstrated in our study. Since, there are currently 248 nuclear research reactors in active operation in over 56 countries throughout the world [28], the accessibility of ^153^Sm should be a viable alternative, reducing the need for expensive international shipping costs.

Since it is proven that styrene DVB copolymer ion exchange resin has a stable chemical structure and function despite the irregular shapes of the microparticles, we are currently looking at the method of spheroidization to produce spherical microparticles which will possess higher mechanical strength and improved flow during intraarterial administration. We were able to achieve 3 GBq of ^153^Sm activity with corresponding number of microparticles similar to that of ^90^Y SIR-Spheres. However, it should be noted that, similar activity of ^153^Sm and ^90^Y will not result in similar therapeutic response because the energies of the beta emissions are different between these two radioisotopes. Hence, further studies to determine ^153^Sm activity equivalent to 3 GBq of ^90^Y, need to be carried out.

Due to the presence of gamma energy from ^153^Sm, radiation exposure received by neighbouring organs and patient’s surrounding need to be considered and a tolerable dose limit needs to be determined. As compared to Iodine-131 (^131^I) treatment with 3.5 fold higher gamma energy and 4 times longer half-life than that of ^153^Sm, protective measures required following radioembolization with ^153^Sm are predicted to be of relatively lesser concern. A dosimetry study for radioembolization with ^166^Ho reported that only 1.1% of the overall absorbed dose were contributed by the gamma emission [29]. Although the contribution by ^153^Sm should be higher due to slightly higher gamma energy and two times longer half-life, the risk is still expected to be minimal as compared to the benefits from dose delivered to the tumour. However, patient’s hospitalisation and crucial radiation monitoring need to be carried out, before the patient can be released following the ^153^Sm treatment. Internal radiation dosimetry on this matter is currently under development using Monte Carlo simulations, which need to be further verified with phantom measurements and animal studies before it can be applied for human administration.

## Conclusion

A theranostic 20–40 μm resin microparticles have been prepared using ion exchange resin (styrene DVB with sulphonic acid group), labelled with ^153^Sm produced via neutron activation. The ^153^Sm-Amberlite microparticles are easy to prepare and the procedure does not involve unnecessary radiation exposure during the labelling process. They were able to withstand prolonged irradiation, at the same time possessed excellent labelling efficiency with strong retention of ^153^Sm tested over 48 h, no radioactive impurities produced from neutron activation, low settling velocity and stable in suspension, and the lower production cost than that of ^90^Y SIR-Spheres. The ^153^Sm-Amberlite microparticles have the potential to be used as an alternative to ^90^Y-microspheres, with added advantage of gamma radiation for imaging of activity distribution following TARE. Dosimetric studies to estimate total ^153^Sm activity needed to deliver equivalent tumour dose and therapeutic response from 3 GBq ^90^Y shall be carried out. Further animal studies for *in vivo* pharmacokinetics and biochemical stability will be required prior to clinical studies.

## List of abbreviations

TARE: transarterial radioembolization
HCC: hepatocellular carcinoma
PLLA: poly (L-lactic acid)
DVB: divinylbenzene
PTS: pneumatic transfer system
RR: rotary specimen rack
FTIR: Fourier Transform Infrared spectroscopy
FESEM: Field Emission Scanning Electron Microscopy
EDX: Energy Dispersive X-ray spectroscopy
MNA: Malaysian Nuclear Agency

## References

[1] Ferlay J, Soerjomataram I, Dikshit R, Eser S, Mathers C, et al (2015) Cancer incidence and mortality worldwide: Sources, methods and major patterns in GLOBOCAN 2012. Int J Cancer 136(5): E359–E386. 10.1002/ijc.29210 25220842

[2] Bruix J, Sherman M, Llovet JM, Beaugrand M, Lencioni R, et al (2001) Clinical management of hepatocellular carcinoma. Conclusions of the Barcelona-2000 EASL Conference. European Association for the Study of the Liver. J Hepatology 35(3): 421–430. 10.1016/s0168-8278(01)00130-1 11592607

[3] BTG International (2013) About TheraSphere®. Canada. Available: http://www.therasphere.com/.

[4] U.S. Food and Drug Administration (FDA) (2002) Medical Devices. Products and Medical Procedures. Device Approvals and Clearance. Recently-Approved Devices. USA. Available: http://www.fda.gov/MedicalDevices/ProductsandMedicalProcedures/DeviceApprovalsandClearances/Recently-ApprovedDevices/ucm083605.htm.

[5] Koch W, Tatsch K (2008) Nuclear medicine procedures for treatment evaluation In: Bilbao J, Reiser M, editors. Liver radioembolization with 90Y microspheres: Springer Berlin Heidelberg pp. 75–91.

[6] Gupta T, Virmani S, Neidt TM, Szolc-Kowalska B, Sato KT, et al (2008) MR tracking of iron-labeled glass radioembolization microspheres during transcatheter delivery to rabbit VX2 liver tumors: feasibility study. Radiology 249(3): 845–854. 10.1148/radiol.2491072027 18840788 PMC6944075

[7] Riaz A, Awais R, Salem R (2014) Side effects of Yttrium-90 radioembolization. Front Oncol 4: 198 10.3389/fonc.2014.00198 25120955 PMC4114299

[8] Qaim SM (2001) Therapeutic radionuclides and nuclear data. Radiochim Acta 89: 297–302.

[9] Yeong CH, Abdullah BJ, Ng KH, Chung LY, Goh KL, et al (2012) Production and first use of 153SmCl3-ion exchange resin capsule formulation for assessing gastrointestinal motility. Appl Radiat Isot 70(3): 450–455. 10.1016/j.apradiso.2011.11.056 22178699

[10] Laboratoire National Henri Becquerel (2014) Recommended data (by Z). France. Available: http://www.nucleide.org/DDEP_WG/DDEPdata_by_Z.htm.

[11] IAEA (1974) Handbook on nuclear activation cross-sections In: Brune D, Schmidt JJ, editors. Technical Reports Series No. 156. Vienna: International Atomic Energy Agency.

[12] Kim DH, Chen J, Omary RA, Larson AC (2015) MRI visible drug eluting magnetic microspheres for transcatheter intra-arterial delivery to liver tumors. Theranostics 5(5): 477–488. 10.7150/thno.10823 25767615 PMC4350010

[13] Wheaton RM, Lefevre LJ (2000) Dow Liquid Separations: DOWEX ion exchange resins. Fundamentals of ion exchange. Form No. 177-177-01837-600QRP: The Dow Chemical Company. Available: http://msdssearch.dow.com/PublishedLiteratureDOWCOM/dh_0032/0901b803800326ca.pdf?filepath=liquidseps/pdfs/noreg/177-01837.pdf&fromPage=GetDoc.

[14] Dow. Dow water and process solutions. Products. AMBERLITETM IR 120 H. The Dow Chemical Company. Available: http://www.dowwaterandprocess.com/en/Products/A/AMBERLITE_IR120_H.

[15] Helfferich FG (1964) Ion Exchange: McGraw-Hill Inc., USA 640 p.

[16] Thermo Fisher Scientific (2009) Derivation of count per milliliter from percentage of solids. Technical Note: TN-017.04. USA. Available at: www.thermo.com/particletechnology

[17] University Malaya Medical Centre (UMMC) (2014) Department of Transfusion Medicine *(Jabatan Perubatan Transfusi)* Introduction: Blood Donation. Kuala Lumpur Available: http://www2.ummc.edu.my/view/departments.php?deptID=16&page=1.

[18] University Malaya Medical Centre (UMMC) Medical Ethics Committee (MEC) (2015) Medical Ethics Application Standard Operating Procedure (SOP) *(Prosedur Kualiti Permohonan Kelulusan Etika Perubatan)*. Document No.: PK-7.1-QSU-011-E02. Kuala Lumpur, Malaysia: University of Malaya Medical Centre pp. 1–19. Available: http://www2.ummc.edu.my/backend/view/storage/files/ethic/SOP%20edited%2004022015%20onlineversibaru%281%29.pdf.

[19] University Malaya Medical Centre (UMMC) (2015) Researcher Research Ethics: UMMC Medical Ethics Committee Kuala Lumpur, Malaysia Available: http://www2.ummc.edu.my/view/content.php?ID=VGxSWlBRPT0=.

[20] Yeong CH, Abdullah BJ, Ng KH, Chung LY, Goh KL, et al (2011) Neutron-activated 153Sm-ion-exchange resin as a tracer for gastrointestinal scintigraphy. Nucl Med Commun 32(12): 1256–1260. 10.1097/MNM.0b013e32834b3ac8 21934547

[21] Singare P, Lokhande R, Madyal R (2011) Thermal degradation studies of some strongly acidic cation exchange resins. Open J Phys Chem 1(2): 45–54.

[22] Mumper RJ, Ryo UY, Jay M (1991) Neutron-activated Holmium-166-poly (L-lactic acid) microspheres: a potential agent for the internal radiation therapy of hepatic tumors. J Nucl Med 32(11): 2139–2143. 1941151

[23] Hruby M, Skodova M, Mackova H, Skopal J, Tomes M, et al (2011) Lutetium-177 and Iodine-131 loaded chelating polymer microparticles intended for radioembolization of liver malignancies. React Funct Polym 71(12): 1155–1159.

[24] Poorbaygi H, Reza Aghamiri SM, Sheibani S, Kamali-Asl A, Mohagheghpoor E (2011) Production of glass microspheres comprising 90Y and 177Lu for treating of hepatic tumors with SPECT imaging capabilities. Appl Radiat Isot 69(10): 1407–1414. 10.1016/j.apradiso.2011.05.026 21723135

[25] Hafeli UO, Casillas S, Dietz DW, Pauer GJ, Rybicki LA, et al (1999) Hepatic tumor radioembolization in a rat model using radioactive Rhenium (186Re/188Re) glass microspheres. Int J Radiat Oncol Biol Phys 44(1): 189–199. 10219814 10.1016/s0360-3016(98)00554-9

[26] Elder DP (2005) Pharmaceutical applications of ion-exchange resins. J Chem Educ 82: 575.

[27] Hafeli UO, Roberts WK, Pauer GJ, Kraeft SK, Macklis RM (2001) Stability of biodegradable radioactive Rhenium (Re-186 and Re-188) microspheres after neutron activation. Appl Radiat Isot 54: 869–879. 11300399 10.1016/s0969-8043(00)00313-4

[28] IAEA (2015) Research Reactor Databases. Research Reactors. International Atomic Energy Agency. Available: http://nucleus.iaea.org/RRDB/RR/ReactorSearch.aspx?filter=0.

[29] Turner JH, Claringbold PG, Klemp PFB, Cameron PJ, Martindale AA, et al (1994) 166Ho-microsphere liver radiotherapy: a preclinical SPECT dosimetry study in the pig. Nucl Med Commun 15(7): 545–553. 7970432

